# Interaction between human leukocyte antigen (HLA-C) and killer cell Ig-like receptors (KIR2DL) inhibits the cytotoxicity of natural killer cells in patients with hepatoblastoma

**DOI:** 10.3389/fmed.2022.947729

**Published:** 2022-11-23

**Authors:** Jing-Jie Guo, Yong-Qin Ye, Yi-Di Liu, Wei-Fang Wu, Qian-Qian Mei, Xi-Yun Zhang, Jing Lao, Bin Wang, Jian-Yao Wang

**Affiliations:** ^1^Shenzhen Children’s Hospital of China Medical University, Shenzhen, Guangdong, China; ^2^Department of General Surgery, Shenzhen Children’s Hospital, Shenzhen, Guangdong, China; ^3^Shenzhen Children’s Hospital of Shantou University Medical College, Shenzhen, Guangdong, China

**Keywords:** hepatoblastoma, single-cell transcriptome sequencing, PBMC, NK cells, KIR2DL

## Abstract

**Background:**

Hepatoblastoma (HB) is the most common liver malignancy in childhood with poor prognosis and lack of effective therapeutic targets. Single-cell transcriptome sequencing technology has been widely used in the study of malignant tumors, which can understand the tumor microenvironment and tumor heterogeneity.

**Materials and methods:**

Two children with HB and a healthy child were selected as the research subjects. Peripheral blood and tumor tissue were collected for single-cell transcriptome sequencing, and the sequencing data were compared and analyzed to describe the differences in the immune microenvironment between children with HB and normal children.

**Results:**

There were significant differences in the number and gene expression levels of natural killer cells (NK cells) between children with HB and normal children. More natural killer cells were seen in children with HB compared to normal control. KIR2DL were highly expressed in children with HB.

**Conclusion:**

Single-cell transcriptome sequencing of peripheral blood mononuclear cells (PBMC) and tumor tissue from children with HB revealed that KIR2DL was significantly up-regulated in NK cells from children with HB. HLA-C molecules on the surface of tumor cells interact with inhibitory receptor KIR2DL on the surface of NK cells, inhibiting the cytotoxicity of NK cells, resulting in immune escape of tumors. Inhibitors of related immune checkpoints to block the interaction between HLA-C and KIR2DL and enhance the cytotoxicity of NK cells, which may be a new strategy for HB treatment.

## Background

As the most common primary liver malignancy in childhood, hepatoblastoma (HB) accounts for more than 90% of liver tumors in children under 5 years old, with an incidence rate of 1.2–1.5/million children (1). In recent years, with the increase in the survival rate of premature infants and low birth weight infants, and the influence of tumor susceptibility, the incidence of HB has increased (2). Children with HB are generally characterized by large abdominal masses and abnormally elevated alpha-fetoprotein (AFP) levels (3). HB is a typical occult cancer that lacks early symptoms. As a result, most patients are diagnosed at a late stage, making surgical removal difficult (4). Therefore, neoadjuvant chemotherapy is required to reduce the tumor size and make surgery possible (5, 6). Neoadjuvant chemotherapy combined with surgical resection is the main treatment. There are two commonly used clinical classification systems for the disease. The PRE-TEXT classification system functions based on the area of the tumor involving the liver before treatment, and it is often used to evaluate the possibility of surgical removal of the tumor. The POST-TEXT classification system evaluates tumor size and involved liver area after neoadjuvant chemotherapy for post-chemotherapy surgery (7, 8). There is not enough understanding of the disease, and there is no effective targeted treatment. Recent studies revealed the relationship between WTAP and ALKBH5 gene single nucleotide polymorphisms and the risk of HB through epidemiological investigation of HB, providing a direction for the study of the occurrence of HB (9, 10).

Since Tang et al. published an article on single-cell transcriptome sequencing in the journal Nature Methods in 2009, single-cell transcriptome sequencing has been widely used in the research of various animals, plants, and microorganisms (11), achieving a leap from tissue to cell. At present, single-cell transcriptome sequencing has also been upgraded to third-generation sequencing, which has been applied in many tumor studies, such as understanding the characteristics of tumor microenvironment in lung cancer patients and colorectal cancer patients (12, 13), breast cancer immune cell phenotypes in tumors (14), the characteristics of renal cancer cells (15). The application of single-cell transcriptome sequencing technology in pediatric HB is rare. We conducted a preliminary exploration, focusing on the changes of NK cells, and found the interaction of HLA-C with inhibitory receptors might be an important mechanism for regulating NK cell activity.

## Materials and methods

### Recruitment of participants and collection of samples

The Ethics Committee of Shenzhen Children’s Hospital have approved this study (Approval No. 2021123). A total of three patients were recruited in this study. No. 1 was a 4-year-old female patient with HB, No. 2 was a 2-year-old female patient with HB, and No. 3 was a 3-year-old normal child (named S1, S2, and S3). 5 mL from peripheral blood was extracted from the enrolled patients and put into EDTA anticoagulant tube to prepare cell suspension of PBMC (16, 17). 1*1*1cm tumor tissue and adjacent liver tissue of surgical patients were also selected as research objects.

### Data processing of single-cell RNA sequencing

The preparation and cell suspension of peripheral blood mononuclear cells (PBMC) were performed in accordance with the protocol of professor Noa Bossel Ben-Moshe (16). After the brief centrifugation of single-cell lysates, the cDNA synthesis and library construction were performed using the 10 × Genomics Single-Cell 3′ Library V2 Kit. Subsequently, the two libraries were sequenced as 100-bp paired-end reads on a DNBSEQ sequencer. The Cell Ranger v5.0.1 software was adopted to process the raw files of FASTQ format. The sequencing reads were aligned to the GRCh38 reference transcriptome using STAR. A filtered UMI expression profile was generated for each cell (18).

10x scRNA-Seq gene expression quantification, is mainly based on UMI counts. Very few genes are usually identified due to low quality or empty droplets. Twin or multicellular droplets may show an unusually high number of genes. Use Seurat v3.0.2 software to filter out cells with less than 200 genes or a maximum number of genes greater than 90%; filter out cells with mitochondrial reads ratio greater than 15%; and correct the effect of cell cycle.

### Cell clustering

We select subsets with highly variable cells from the entire dataset (that is, they are strongly expressed in some cells but not in others) and focusing on these genes in the downstream analysis allows us to emphasize the biological signals in the single cell dataset. By default, the 2,000 genes with the greatest degree of variation are chosen. These genes will be utilized in further studies such as PCA. The single-cell data were analyzed by the Seurat v3.0.2 package (19).

The principal components were used for cluster identification using a uniform manifold approximation and projection (UMAP) algorithm. For each cluster, the marker genes were identified using the FindConservedMarkers function as implemented in the Seurat package (logfc. threshold > 0.25 and minPct > 0.25). Subsequently, clusters were remarked to a known cell type according to the MonacoImmuneData database and CellMark database (20, 21).

### Gene differential expression and functional enrichment analysis

Differently expressed genes (DEGs) across various samples or clusters were identified using the FindConservedMarkers function in Seurat with following parameters: logfc. threshold > 0.25, minPct > 0.25, and Padj ≤ 0.05. The phyper function in R program was used to do gene ontology (GO) analysis and kyoto encyclopedia of genes and genomes (KEGG) pathway analysis, and FDR = 0.05 was regarded substantially enriched (22, 23).

## Results

### Clinical symptoms and laboratory data

Both S1 and S2 presented as an abnormal abdominal mass, and ultrasound showed a large hepatic space-occupying lesion with an abnormally elevated AFP level (Table 1). Combined with clinical indications, a primary diagnosis of HB (PRE-TEXT IV) was made. Neither S1 nor S2 have taken any drugs or other treatment regimens when blood samples were collected. HB was further confirmed from pathological results. S3 was a 3-year-old normal child as a healthy control.

**TABLE 1:** Blood result of hepatoblastoma (HB) patients and normal control.

	S1	S2	S3
Leukocyte counts (10^9/L, 5–12)	6.52	9.92	13.52
Neutrophile granulocyte counts (10^9/L, 2–7)	3.48	8.38	3.36
Lymphocytes counts (10^9/L, 0.8–4)	2.57	1.34	8.92
Monocyte counts (10^9/L, 0.12–1)	0.3	0.17	0.72
Eosinophil granulocyte counts (10^9/L, 0.02–0.5)	0.13	0.01	0.47
Basophil granulocyte counts (10^9/L, 0–0.1)	0.04	0.02	0.05
Neutrophile granulocyte (%, 50–70)	53.4	84.5	24.8
Lymphocytes (%, 20–40)	39.4	13.5	66
Monocyte (%, 3–10)	4.6	1.7	5.3
Eosinophil granulocyte (%, 0.5–5)	2	0.1	3.5
Basophil granulocyte (%, 0–1)	0.6	0.2	0.4
Red blood cell (10^12/L, 3.5–5.5)	3.99	2.33	5.15
Hemoglobin (g/L, 110–160)	99	56	140
Platelets counts (10^9/L, 100–300)	916	808	441
C-reactive protein (mg/L, 0–10.0)	3.38	18	NA
AFP (ng/ml, 2.88–20.94)	1,289,089	754,031	NA

Children with HB had higher absolute neutrophil value and ratio of neutrophils than normal children. Hemoglobin levels were low while platelet levels were high, with varying degrees of anemia and hypercoagulable states (Table 1).

### Single-cell transcription atlas of peripheral blood mononuclear cells

**FIGURE 1:**
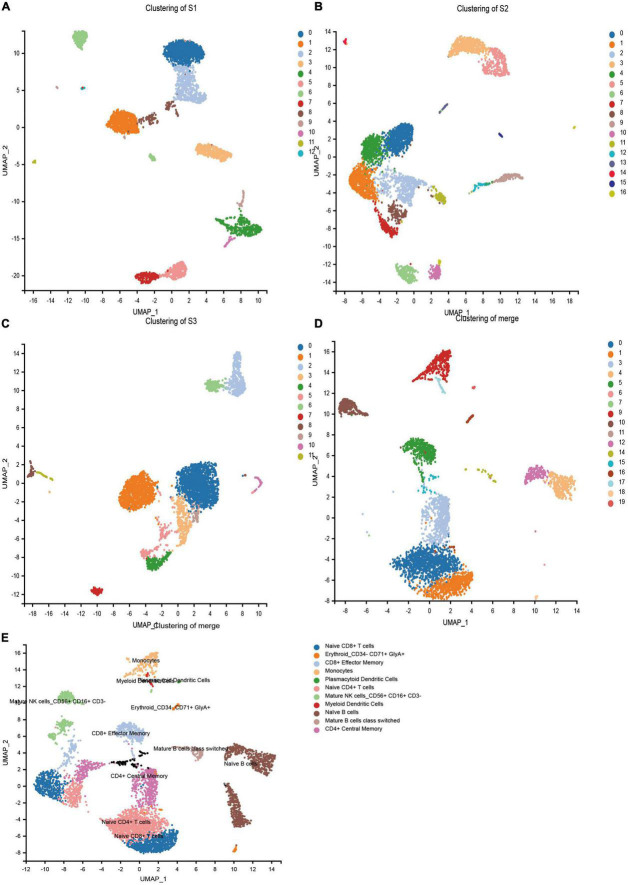
Cell types identified in peripheral blood mononuclear cells (PBMCs) by uniform manifold approximation and projection (UMAP). **(A)** 13 cell clusters in S1 (*n* = 9,014). **(B)** 17 cell clusters in S2 (*n* = 8,178). **(C)** 12 cell clusters in S3 (*n* = 8,615). **(D)** 20 cell clusters in three samples merged (*n* = 25,807). **(E)** 20 cell clusters in three samples (*n* = 25,807), eleven cell types were identified.

In order to obtain the transcriptome information of PBMC from children with HB, the single-cell transcriptome sequencing was performed using the 10 × Chromium platform. Transcriptomic information of 11,594, 10,608, and 11,225 cells were obtained from S1, S2, and S3, respectively. On average, 1,812, 1 478, and 1,517 genes were detected per cell, respectively. After strict quality control and data filtering (see methods section), 9,014, 8,178, and 8,615 cells (Table 2) were finally obtained. Based on principal component analysis (PCA), the UMAP algorithm was used to cluster cells with similar expression patterns through dimensionality reduction. The three samples were divided into 12, 16, and 11 cell clusters (Figures 1A–C). After the combination of the three samples, 20 cell clusters (Figure 1D) were obtained. According to the marker genes of each cell in the sample, the cells were identified (reference database is MonacoImmuneData database). A total of 11 cell types were identified (Figure 1E).

**TABLE 2:** Statistical tables of sample cells and gene expression.

Sample name	S1	S2	S3	S1A	S1B
Estimated number of cells	11,594	10,608	11,225	10,518	7,662
Fraction reads in cells	93.00%	82.10%	90.70%	93.70%	88.20%
Mean reads per cell	63,644	40,407	64,238	75,616	95,867
Median genes per cell	1,812	1,478	1,517	2,704	1,497
Total genes detected	24,658	22,599	23,898	29,234	26,750
Median UMI counts per cell	5,850	5,102	5,061	9,749	3,828
Filtered min count cells	11,464	10,394	11,167	10,483	7,580
Filtered doublet cells	10,611	9,634	10,142	10,334	7,476
Filtered min feature cells	10,606	9,623	10,137	10,327	7,473
Filtered mt proportion	9,014	8,178	8,615	8,776	6,351
Final Cell Num	9,014	8,178	8,615	8,776	6,351

**FIGURE 2:**
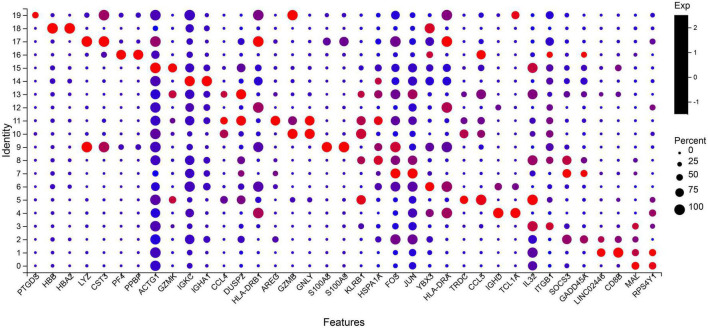
Select the two most significant genes in each cluster to display in the bubble plot. Dot size indicates the proportion of cells in cluster expressing each marker gene, and color indicates the relative expression level (low to high reflected as blue to red). Top two marker genes are ordered as cluster 19 (*PTGDS* and *HBB*) to cluster 0 (*MAL* and *RPS4Y1*). Naive CD4 + T cells: 0, 7; Naive CD8 + T cells: 1, 2; CD4 + Central Memory: 3, 8; CD8 + Effector Memory: 5, 13; Naïve B cells: 4, 6, 12; Mature B cells class switched: 14; Monocytes: 9; Mature NK cells_CD56 + CD16 + CD3-: 10, 11, 19; Erythroid_CD34- CD71 + GlyA +: 16, 18; Myeloid Dendritic Cells: 17; NA: 15.

The two most abundant marker genes in each cluster were selected and illustrated with dot plots across the three samples (Figure 2). There are only 37 marker genes instead of 40 because the three clusters share some marker genes. The marker genes *LYZ*, *DUSP2*, and *GNLY* are shared by clusters 9 and 17, 11 and 13, and 10 and 11, respectively.

### Differences in immune cell subsets among the three children

No more specific cell groups were found in the three samples. We counted the number of cells in the main group (Table 3), S3 as healthy control, the main cell types in the top five were Naive CD4 + T cells, Naive CD 8 + T cells, naïve B cells, CD4 + Central Memory, and CD8 + Effector Memory. Compared with S3, both S1 and S2 showed a higher proportion of NK cells, while the proportion of Naive CD4 + T cells decreased and the proportion of CD4 + Central Memory increased. In conclusion, there are significant cell subset differences between HB patients and normal children.

**TABLE 3:** The cell number of main cluster.

	S1	S2	S3
Mature NK cells_CD56 + CD16 + CD3-	636	773	192
Naive CD4 + T cells	2,280	1,161	3,512
Naive CD8 + T cells	1,525	2,309	2,044
CD4 + Central memory	1,359	1,010	621
CD8 + Effector memory	947	630	653
Naïve B cells	1,069	1,533	1,294
Mature B cells class switched	51	329	76
Monocytes	789	55	116

### GO and KEGG enrichment analysis of differentially expressed genes in NK cells

**FIGURE 3:**
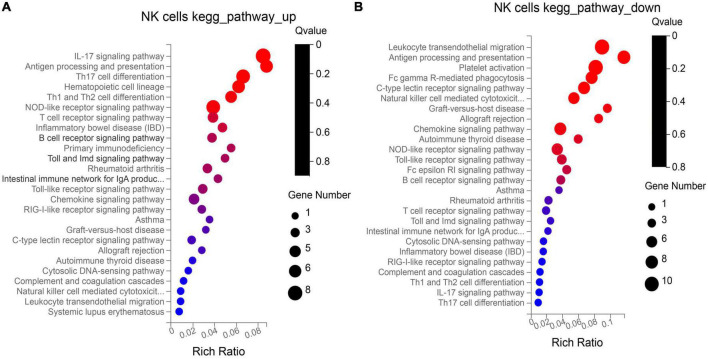
Kyoto encyclopedia of genes and genomes (KEGG) enrichment analysis of up-regulation **(A)** and down-regulation **(B)** genes in NK cells.

**FIGURE 4:**
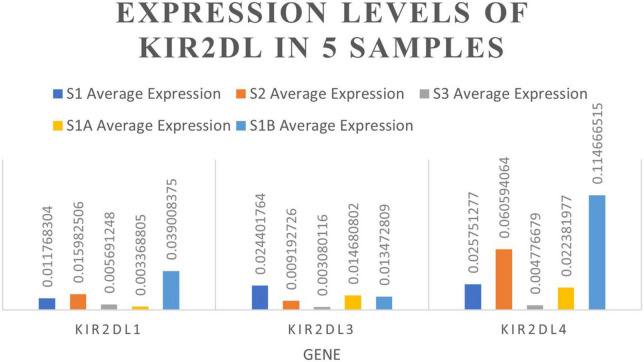
Differences in the expression levels of KIR2DL1, KIR2DL3, and KIR2DL4 among five samples.

NK cells function as tumor killer cells, and the changes are obvious in HB patients. In order to further study the difference of gene level, we analyzed the differential expression of NK cells from two HB patients and one normal control (10, 11, 19 cluster). A total of 310 DEGs were obtained. Among them, there were 122 up-regulated genes and 188 down-regulated genes. The DEGs were subjected to KEGG and GO enrichment analysis (Supplementary Table 1), of which 122 up-regulated genes were enriched to 26 immune–related pathways (Figure 3A), and 188 down-regulated genes were also enriched to 26 immune-related pathways (Figure 3B). Among the up- regulated genes, there are six genes related to antigen processing and presentation (*HSP90AA1*, *HSP90AB1*, *HSPA8*, *HLA-DQB1*, *KIR2DL4*, *HSPA1A*), among which the average expression of KIR2DL4 was more significant (Figure 4), which was closely related to the natural killing effect of NK cells.

### Gene set enrichment analysis of NK cells from three samples

**FIGURE 5:**
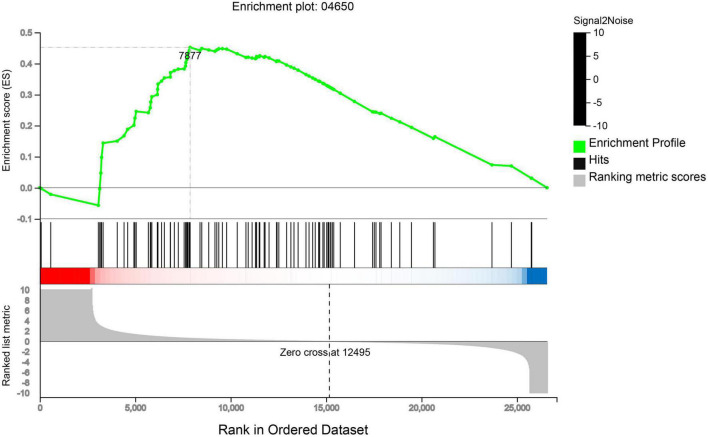
Gene set enrichment analysis (GSEA) analysis of NK cell gene sets in three samples. The first part: The green line at the top is the line graph of the gene Enrichment Score. The vertical axis is the corresponding running ES, and there is a peak in the line graph. The peak is the enrichment score of the gene set, and the gene before the peak is the core gene under the gene set. The horizontal axis represents each gene under this gene set, corresponding to the vertical bar-like bar in the second part. The second part: the barcode-like part is Hits, and each vertical line corresponds to a gene under the gene set. The third part: is the rank value distribution map of all genes, and the ordinate is the ranked list metric, that is, the value of the ranking amount of the gene, which can be understood as “the fold change value after formula processing”.

Gene Set Enrichment Analysis (GSEA) analysis was performed on the NK cell gene sets of the three blood samples. 96 genes were identified to be related to NK cell-mediated cytotoxicity, including 34 core genes (Supplementary Table 2). These core genes were all located on the left side of the peak (leading edge subset) and were all over-expressed in HB (Figure 5). Among them, KIR2DL1, KIR2DL3, and KIR2DL4 play a significant role in the pathway and inhibit the cytotoxic effect of NK cells.

### Single-cell transcriptome analysis of tumor tissue and paracancerous liver tissue

**FIGURE 6:**
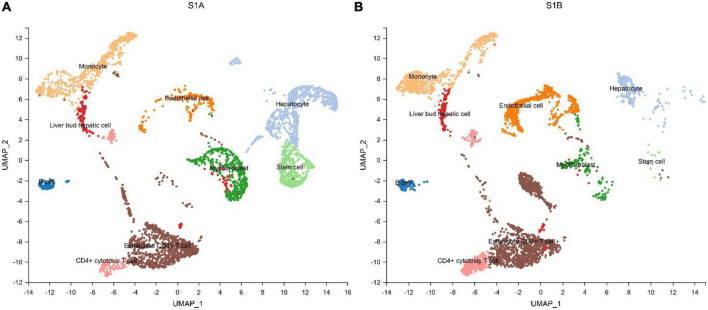
Cell types identified in tumor tissue **(A)** and paracancerous liver tissue **(B)** by uniform manifold approximation and projection (UMAP).

We further performed single-cell transcriptome sequencing study on tumor tissues and paracancerous liver tissues of patients with hepatoblastoma. Patients S1 underwent tumor resection before chemotherapy, and samples that met the research requirements were collected, including tumor tissue samples (S1A) and paracancerous liver tissue samples (S1B); patient S2 underwent ultrasound-guided puncture, and the obtained sample was excluded from the calculation of cell viability. Transcriptomic information of 10,518 and 7,662 cells were obtained from S1A and S1B, respectively. On average, 2,704 and 1,497 genes were detected per cell, respectively. After strict quality control and data filtering, 8,776 and 6,351 cells (Table 2) were finally obtained. Based on PCA, the UMAP algorithm was used to cluster cells with similar expression patterns through dimensionality reduction. According to the marker genes of each cell in the sample, the cells were identified (reference database is CellMark). Nine cell types were identified in S1A and S1B each, and no specific cell groups were found (Figure 6). Tumor tissue contained a higher proportion of stem cells and myofibrillar cells but fewer liver cells than paracancerous liver tissue. Endothelial cells occupied in a higher proportion in both tissue samples. The immune cells were mainly monocytes and CD4 + T cells. We further analyzed the average expression levels of KIR2DL1, KIR2DL3, and KIR2DL4 in all samples (Figure 4). Compared with normal children, KIR2DL was significantly over-expressed in both peripheral blood samples and tissue samples.

## Discussion

Hepatoblastoma is the most common liver malignancy in children under 5 years of age and is usually presented as an abnormal abdominal mass. Imaging examinations show abnormal space-occupying lesions in the liver, which are generally larger in size. Laboratory tests are characterized by abnormally elevated AFP levels. Most of the children missed an early diagnosis and were already in PRETEXT III/IV stage, and the chance for complete resection in one-stage surgery was slim. Neoadjuvant chemotherapy combined with surgical resection is the diagnosis and treatment plan for most children (24, 25). Due to the lack of understanding of the pathogenesis, there are currently no effective therapeutic targets and immunotherapy options.

We enrolled three age-matched children in our study, and identified that those children with HB had a higher proportion of neutrophils. Neutrophils are the most abundant circulating white blood cells and mediate immune defense through a variety of mechanisms. In the tumor microenvironment, due to the persistent inflammatory response, activated neutrophils play an anti-tumor or tumor-promoting role (26, 27, 28). Meanwhile, children with HB showed different degrees of anemia and coagulation dysfunction. Anemia is the most common hematological manifestation in cancer patients, and the main causes are inflammatory anemia and nutritional anemia (29). The hypercoagulable state created by platelets in the tumor microenvironment also promotes tumor progression and is a new target for tumor therapy (30).

Compared with the healthy control, HB children have a higher proportion of NK cells, this is because the occurrence of tumors stimulates the body’s own immune killing effect, and the body produces more NK cells in compensation. At the same time, the tumor stimulates the differentiation of Naive CD4 + T cells to produce more CD4 + Central Memory, which participates in the immune response of the tumor.

We focused on analyzing the changes of NK cells. NK cells are important immune cells in the body. Unlike T cells and B cells, NK cells have no major histocompatibility complex (MHC) restriction and are independent of antibodies. NK cells belong to lymphocytes that can non-specifically kill tumor cells and virus-infected cells without prior sensitization, so NK cells are an important immune factor in the body’s anti-tumor and anti-infection (31). The indiscriminate killing effect of NK cells on tumor cells makes them an important cell for the study of tumor immunotherapy. The activity of NK cells is co-regulated by activating and inhibiting receptors expressed on the cell surface. KIR2DL is an inhibitory receptor, and tumor cells expressing HLA-C interact with KIR2DL and thus the activity of NK cells can be inhibited. Therefore, blocking the interaction between HLA molecules and inhibitory receptors is an important strategy to enhance the activity of NK cells and improve the anti-tumor ability.

Single-cell transcriptome sequencing of peripheral blood and tumor tissue samples from HB patients were further performed, and it was found that KIR2DL was significantly up-regulated in HB patients. We believe that KIR2DL is highly expressed in the tumor microenvironment of HB. We further studied the signaling pathway of KIR2DL and found that HLA-C molecules interacted with KIR2DL to inhibit the cytotoxicity of NK cells (KEGG pathway: map04650), which may be an important mechanism of HB-mediated immune escape of NK cells.

Human leukocyte antigen (HLA)-C is a class of classical MHC-I molecules whose main function is to bind to inhibitory receptors on the surface of NK cells. HLA-C can be distributed in normal cells, stimulated cells and tumor cells, and is the presenting molecule of endogenous antigens. The specific amino acid sequence on its peptide chain constitutes antigen specificity. Normal cells bind to inhibitory receptors through MHC-I molecules, evade NK cell-dependent cytotoxicity, achieve “self-recognition”, and ensure the stability of the human immune system. Tumor cells can also inhibit NK cell cytotoxicity through the binding of HLA-C molecules to inhibitory receptors, resulting in immune escape (32).

KIR2DL belongs to an atypical killer cell Ig-like receptors (KIRs) family member and it is an inhibitory receptor expressed on the surface of human NK cells. Tumor cells bind to the NK cell surface receptor KIR2DL *via* HLA-C molecules, and activate phosphorylation of tyrosine residues in the cytoplasmic ITIM sequence, leading to the recruitment of protein tyrosine phosphatases (PTPs) SHP-1 and SHP-2. Recruitment and activation of SHP-1/2 have been shown to negatively regulate NK cell killing ability (33). Both SHP-1 and SHP-2 exist in many tumor-related cell signaling pathways, such as in the T cells receptor signaling pathway. The production of SHP-1 is stimulated by PD-1, which mediates negative regulation, shorten the process of cell cycle, inhibits the production of IL-2, inhibits the activation of T cells and subsequently the generation of effector T cells, also promotes the apoptosis of T cells. Inhibitory drugs against the PD–1 immune checkpoint have been widely used for cancer treatment, and inhibitors against the PTP immune checkpoint are also under development (34).

In summary, tumor cells interact with inhibitory receptors on the surface of NK cells through HLA-C molecules, which negatively regulate the cytotoxicity of NK cells. Through our research, we found that the inhibitory receptors on the surface of NK cells in HB patients were significantly overexpressed, which may be the mechanism by which tumor cells regulate the anti-tumor activity of NK cells and lead to immune escape. Blocking the interaction between HLA-C and KIR2DL receptors is an important strategy to enhance NK cell cytotoxicity, which has been reported in related studies (35).

## Limitations

Our study was the first investigation uncovering the immune landscape of HB at the single-cell level. Due to the small number of cases of hepatoblastoma, we studied only two patients and one healthy control. We will include more subjects in the future.

## Conclusion

Through single-cell transcriptome sequencing of PBMC and tumor tissue cells of children with HB, focusing on the differentially expressed genes of NK cells, it was found that the inhibitory receptor KIR2DL on the surface of NK cells was significantly overexpressed. In hepatoblastoma, the interaction between HLA-C molecules and KIR2DL4 may be the mechanism by which tumor cells regulate the antitumor activity of NK cells, leading to tumor immune escape. Developing inhibitors of related immune checkpoints to block the interaction between HLA-C and KIR2DL and enhance the antitumor activity of NK cells may be a new strategy for HB treatment.

## Data availability statement

The data presented in this study are deposited in the China National GeneBank Nucleotide Sequence Archive repository, accession number: CNP0003042. http://db.cngb.org/cnsa/project/CNP0003042_4b985eb5/reviewlink/.

## Ethics statement

The studies involving human participants were reviewed and approved by the Ethics Committee of Shenzhen Children’s Hospital. The patients/participants provided their written informed consent to participate in this study. Written informed consent to participate in this study was provided by the participants’ legal guardian/next of kin. Written informed consent was obtained from the minor(s)’ legal guardian/next of kin for the publication of any potentially identifiable images or data included in this article.

## Author contributions

BW and J-YW designed the study. J-JG wrote the draft. Y-QY, Y-DL, W-FW, Q-QM, JL, and X-YZ conducted the experiments. All authors read and approved the final manuscript.
